# Size-Fractionated Particle Number Concentrations and Daily Mortality in a Chinese City

**DOI:** 10.1289/ehp.1206398

**Published:** 2013-08-13

**Authors:** Xia Meng, Yanjun Ma, Renjie Chen, Zhijun Zhou, Bingheng Chen, Haidong Kan

**Affiliations:** 1 School of Public Health, Key Lab of Public Health Safety of the Ministry of Education, Fudan University, Shanghai, China; 2 Research Institute for the Changing Global Environment and Fudan Tyndall Centre, Fudan University, Shanghai, China; 3 Shenyang Institute of Atmospheric Environment, China Meteorological Administration, Shenyang, China; 4 Shanghai Key Laboratory of Atmospheric Particle Pollution and Prevention (LAP^3^), Fudan University, Shanghai, China; * These authors contributed equally to this work.

## Abstract

Background: Associations between airborne particles and health outcomes have been documented worldwide; however, there is limited information regarding health effects associated with different particle sizes.

Objectives: We explored the association between size-fractionated particle number concentrations (PNCs) and daily mortality in Shenyang, China.

Methods: We collected daily data on cause-specific mortality and PNCs for particles measuring 0.25–10 μm in diameter between 1 December 2006 and 30 November 2008. We used quasi-Poisson regression generalized additive models to estimate associations between PNCs and mortality, and we used natural spline smoothing functions to adjust for time-varying covariates and long-term and seasonal trends.

Results: Mean numbers of daily deaths were 67, 32, and 7 for all natural causes, cardiovascular diseases, and respiratory diseases, respectively. Interquartile range (IQR) increases in PNCs for particles measuring 0.25–0.50 μm were significantly associated with total and cardiovascular mortality, but not respiratory mortality. Effect estimates were larger for PNCs during the warm season than the cool season, and increased with decreasing particle size. IQR increases in PNCs of 0.25–0.28 μm, 0.35–0.40 μm, and 0.45–0.50 μm particles were associated with 2.41% (95% CI: 1.23, 3.58%), 1.31% (95% CI: 0.52, 2.09%), and 0.45% (95% CI: 0.04, 0.87%) higher total mortality, respectively. Associations were generally stable after adjustment for mass concentrations of ambient particles and gaseous pollutants.

Conclusions: Our findings suggest that particles < 0.5 μm in diameter may be most responsible for adverse health effects of particulate air pollution and that adverse health effects may increase with decreasing particle size.

Citation: Meng X, Ma Y, Chen R, Zhou Z, Chen B, Kan H. 2013. Size-fractionated particle number concentrations and daily mortality in a Chinese city. Environ Health Perspect 121:1174–1178; http://dx.doi.org/10.1289/ehp.1206398

## Introduction

Particulate matter (PM) consists of discrete particles that range in size over several orders of magnitude, including inhalable particles (< 10 μm in aerodynamic diameter, PM_10_), coarse particles (PM_2.5–10_), fine particles (PM_2.5_), and ultrafine particles (UFPs; PM < 0.1 μm in aerodynamic diameter). Numerous epidemiologic studies have reported significant positive associations between particulate air pollution and adverse health outcomes (Brunekreef and Forsberg 2005; Chen et al. 2011, 2012; Dominici et al. 2006), but information on health effects according to particle size is limited (Wichmann et al. 2000).

Smaller particles (e.g., ≤ 2.5 μm) are more likely than larger particles to be produced by fuel combustion and to be formed by secondary reactions. Compared with larger particles, small particles have higher total particle number concentrations (PNCs, reported as particles per cubic meter), and they tend to absorb more toxic components, have higher deposition efficiency in the respiratory tract, and have larger surface areas (Delfino et al. 2005; Valavanidis et al. 2008). However, previous findings regarding the health effects of increases in mass concentrations (milligrams per cubic meter) of PM_2.5_ and PM_2.5–10_ have been inconsistent (Brunekreef and Forsberg 2005). Epidemiologic evidence regarding associations with PNCs is inadequate because of the limited availability of size-resolved measurements, and findings also have been inconsistent (Ibald-Mulli et al. 2002). For instance, Pekkanen (et al. 2002) reported significant positive associations of ST-segment depression during exercise tests with PNCs of smaller particles (PNC_0.1–1.0_ and PNC_< 0.1_), with mass concentrations of PM_2.5_, but not PM_2.5–10_. In contrast, Andersen et al. (2008) reported that cardiovascular hospital admissions in the elderly were positively associated with PM_10_ and PNC_0.1–1.0_, but not PNC_< 0.1_.

Most previous studies of PNCs and adverse health outcomes have been conducted in developed countries. Few studies on size-fractionated PNCs have been conducted in China, where particulate air pollution is exceptionally high and may differ from air pollution in developed countries with regard to particle size distributions, chemical components, and other characteristics (Kan et al. 2009). The objective of the present study was to explore the association between short-term exposures to size-fractionated PNCs and daily mortality in Shenyang, China.

## Methods

*Data*. Shenyang is the capital city of Liaoning province and the largest city in northeastern China. Our study area was limited to the urban areas of Shenyang and had a target population of 3.5 million by 2008. The major sources of PM air pollution in Shenyang are coal combustion, urban traffic emissions, building construction, the chemical industry, and natural dust.

We obtained daily mortality data for urban residents of Shenyang between 1 December 2006 and 30 November 2008 (731 days) from the Liaoning Provincial Center for Disease Control and Prevention. The causes of death were coded according to the *International Classification of Diseases, Revision 10* (ICD-10; World Health Organization 1992) and categorized as deaths due to nonaccidental causes (ICD-10 codes A00–R99), cardiovascular disease (ICD-10 codes I00–I99), and respiratory disease (ICD-10 codes J00–J98).

An automatic continuous monitoring system was installed on the rooftop of the Shenyang Regional Meteorological Centre to measure daily PNCs with size distributions between 0.25 μm and 10 μm. Shenyang Regional Meteorological Centre is located in Shenhe District and is mainly surrounded by residential and commercial areas. In accord with Chinese government regulations, the monitor was located away from major roads, industrial sources, buildings, and residential sources of emissions from burning coal, waste, or oil. The sampling instrument was placed 10 m above ground. Aerosol number size distributions of particles between 0.25 μm and 10 μm were measured continuously with an Ambient Dust Monitor 365 (GRIMM; Grimm Aerosol Technik GmbH & Co. KG, Ainring, Germany), which uses light-scattering technology to measure PNCs.

Particle diffusion is the primary mechanism for the deposition of particles ≤ 0.5 μm in the respiratory tract, in contrast with particles between 0.5 and 2.5 μm that are primarily deposited by sedimentation (Wichmann et al. 2000). Therefore, we calculated daily mean PNCs for particles categorized according to multiple size fractions, specifically, PNC_0.25–0.28_, PNC_0.28–0.30_, PNC_0.30–0.35_, PNC_0.35–0.40_, PNC_0.40–0.45_, PNC_0.45–0.50_, PNC_0.50–0.65_, PNC_0.65–1.0_, PNC_1.0–2.5_, and PNC_2.5–10_. We also collected daily average mass concentrations of PM_2.5_, PM_10_, PM_2.5–10_, sulfur dioxide (SO_2_), and nitrogen dioxide (NO_2_) at the same station. The optical scattering method was used to measure mass concentrations of PM_2.5_ and PM_10_ (GRIMM Environmental Dust Monitor, EDM 180), and PM_2.5–10_ concentrations were estimated by subtracting PM_2.5_ from PM_10_ concentrations. Methods based on ultraviolet ﬂuorescence and chemiluminescence (models 43A and 42C, respectively; Thermo Environmental Instruments Inc., Franklin, MA, USA) were used to measure SO_2_ and NO_2_. Twenty-four–hour average concentrations of PM_10_, PM_2.5_, SO_2_, and NO_2_ were collected for all days with data for ≥ 75% of all 1-hr measurements on that day. Meteorological data (daily mean temperature and relative humidity) were obtained from the Shenyang Meteorological Bureau. The institutions that provided mortality, air pollution, and weather data all currently participate in detailed quality assurance and quality control programs mandated by the Chinese government.

*Statistical analysis*. We used a time-series design to investigate the short-term effects of size-fractioned PNCs on daily mortality (Zeger et al. 2006). Specifically, we used generalized additive models (GAMs) with quasi-Poisson regression (Bell et al. 2004). The same analytical protocol has been used in the Public Health and Air Pollution in Asia (PAPA) project (Wong et al. 2008) and the China Air Pollution and Health Effects Study (CAPES) (Chen et al. 2012). The GAMs incorporated natural cubic smooth functions of calendar time with 7 degrees of freedom (df) per year to control for long-term and seasonal trends in daily mortality. We also controlled for the current-day mean temperature and relative humidity using natural cubic smooth functions with 6 and 3 df, respectively (Chen et al. 2012), and included an indicator variable for the day of the week in our model.

Consistent with previous studies, we used 2-day moving average PNCs for the current and previous day (lag 01) in our main analyses (Chen et al. 2012; Wong et al. 2008). In addition to all-year analyses, we performed separate analyses for the warm season (May–October) and cool season (November–April) to assess the potential modifying role of season.

We performed several sensitivity analyses to examine the robustness of our findings. First, we fitted separate two-pollutant models adjusted for mass concentrations of PM_10_, PM_2.5_, PM_2.5–10_, SO_2_, or NO_2_, respectively. Second, we estimated effects using different lag structures, including single-day lags (from lag 0 for the current day to lag 5 for the 24-hr average exposure 5 days prior to the current day) and the 6-day moving average concentration for the current day and the previous 5 days (lag 05). In addition, we evaluated the impacts of using alternative df values for the smooth time trend function, of adjusting for temperature using a 3-day moving average temperature (lag 1–3) to account for any delayed effects of temperature on daily mortality, and of estimating associations with broader particle size categories (PNC_0.25–0.3_, PNC_0.3–0.5_, and PNC_0.5–1.0_).

All analyses were conducted in R 2.15.1 using the MGCV package (version 1.7–22; R Foundation for Statistical Computing, Vienna, Austria). Statistical tests were two-sided, and *p*-values of ≤ 0.05 were considered statistically significant. The results are presented as mean percent changes in daily mortality [with 95% confidence intervals (CIs)] associated with an interquartile range (IQR) increase in size-fractionated PNCs. For seasonal analyses, exposure contrasts were scaled to IQRs for season-specific exposure distributions.

## Results

During the study period, the mean numbers of daily deaths in Shenyang were 67, 32, and 7 for deaths due to all natural causes, cardiovascular diseases, and respiratory diseases, respectively (Table 1). The overall PNC for particles from 0.25 to 10 μm in diameter was dominated by particles ≤ 0.35 μm, which accounted for 84% of the total PNC. In contrast, coarse particles (PM_2.5–10_) accounted for < 0.1% of the total PNC. For each size class, PNCs were higher in the cool season than in the warm season (see Supplemental Material, Table S1).

**Table 1:** Summary statistics of daily death numbers, air pollution levels, and weather conditions in Shenyang, China.

Variable	Mean ± SD	Minimum	Maximum	IQR
Daily deaths (*n*)
All natural causes	67 ± 10	41	113	14
Cardiovascular	32 ± 7	15	76	9
Respiratory	7 ± 3	1	18	4
Air pollutant^*a*^
PNC_0.25–10_	11,000 ± 400	1,100	71,000	8,400
PNC_0.2__5–0__.28_	3,500 ± 2,700	380	16,000	2,600
PNC_0.2__8–0__.30_	2,700 ± 2,300	260	15,000	2,000
PNC_0.3__0–0__.35_	1,900 ± 1,800	170	13,000	1,510
PNC_0.3__5–0__.40_	1,100 ± 1,300	81	9,600	850
PNC_0.4__0–0__.45_	520 ± 810	28	6,400	360
PNC_0.4__5–0__.50_	290 ± 520	11	4,300	193
PNC_0.5__0–0__.65_	270 ± 21	9.2	4,300	188
PNC_0.65–1.0_	110.0 ± 8.9	3.8	2,000	63
PNC_1.__0–2__.5_	32.0 ± 3.5	1.0	1,900	22
PNC_2.5–10_	9.40 ± 4.2	0.1	2,600	4
PM_2.5_	95.9 ± 56.0	11.0	424.0	63.9
PM_2.5–10_	48.0 ± 37.5	3.5	573.2	36.7
PM_10_	142.5 ± 72.9	18.1	703.6	86.3
SO_2_	55.2 ± 44.9	8.0	331.0	53.0
NO_2_	35.3 ± 16.9	9.0	108.0	19.0
Weather condition
Temperature (°C)	8.8 ± 12.3	–18.0	27.0	23.0
Humidity (%)	65.2 ± 14.1	16.0	98.0	20.0
^***a***^PNC are reported as particles/cm^3^, and mass concentrations of PM, SO_2_, and NO_2_ are reported as μg/m^3^.				

Generally, PNCs for particles ≤ 2.5 μm in diameter were strongly correlated with mass concentrations of PM_2.5_, and moderately correlated with mass concentrations of PM_10_, PM_2.5–10_, SO_2_, and NO_2_ (Table 2). In contrast, these pollutants were not correlated with PNCs for particles > 2.5 μm. PNCs for particles ≤ 2.5 μm were moderately correlated with temperature and humidity on the same day (Table 2).

**Table 2:** Correlation coefficients for daily mean values of meteorologic and air pollution variables.

Particle fraction	Temperature (°C)	Humidity (%)	PM_2.5 _(μg/m^3^)	PM_10 _(μg/m^3^)	PM_2.5–10_(μg/m^3^)	SO_2 _(μg/m^3^)	NO_2 _(μg/m^3^)
PNC_0.2__5–0__.28_	–0.32	0.22	0.70	0.55	0.05	0.54	0.48
PNC_0.2__8–0__.30_	–0.32	0.23	0.71	0.57	0.07	0.54	0.47
PNC_0.3__0–0__.35_	–0.30	0.24	0.71	0.57	0.08	0.52	0.47
PNC_0.3__5–0__.40_	–0.28	0.23	0.65	0.54	0.11	0.50	0.46
PNC_0.4__0–0__.45_	–0.28	0.19	0.60	0.52	0.15	0.48	0.44
PNC_0.4__5–0__.50_	–0.29	0.17	0.57	0.51	0.18	0.47	0.43
PNC_0.5__0–0__.65_	–0.29	0.15	0.55	0.51	0.19	0.47	0.42
PNC_0.65–1.0_	–0.32	0.11	0.52	0.50	0.23	0.46	0.38
PNC_1.__0–2__.5_	–0.21	0.01	0.25	0.27	0.16	0.29	0.21
PNC_2.5–10_	–0.08	0.04	0.02	0.03	0.3	0.10	0.03

In the all-year analyses, PNCs of 0.25–0.50 μm particles were significantly associated with total and cardiovascular mortality, with stronger associations as particle sizes decreased (Table 3). For example, IQR increases in PNC_0.25–0.28_, PNC_0.35–0.40_, and PNC_0.45–0.50_ were associated with 2.41% (95% CI: 1.23, 3.58%), 1.31% (95% CI: 0.52, 2.09%), and 0.45% (95% CI: 0.04, 0.87%) higher daily average values for total mortality, respectively. Associations were strongest for cardiovascular mortality, whereas corresponding associations with respiratory mortality were weaker and not statistically significant for PNCs of any size fractions. We also estimated significant positive associations with IQR and 10-μg/m^3^ increases in mass concentrations of PM_2.5_ (with all-cause and cardiovascular mortality) and PM_10_ (for all three mortality outcomes), but not PM_2.5–10_ (see Supplemental Material, Table S2).

**Table 3:** Percent change (95% CI) of daily mortality associated with a 2-day moving average IQR incremental change in PNCs in Shenyang, China.

Particle fraction	All natural causes			Cardiovascular			Respiratory		
	All period	Warm^*a*^	Cool^*b*^	All period	Warm^*a*^	Cool^*b*^	All period	Warm^*a*^	Cool^*b*^
PNC_0.2__5–0__.28_	2.41 (1.23, 3.58)*	4.21 (2.43, 5.99)*	1.92 (–0.14, 3.97)	2.79 (1.09, 4.49)*	4.58 (1.91, 7.27)*	2.17 (–0.77, 5.10)	0.72 (–2.97, 4.40)	3.84 (–2.00, 9.68)	1.06 (–5.31, 7.43)
PNC_0.2__8–0__.30_	2.10 (1.03, 3.18)*	4.06 (2.25, 5.86)*	1.74 (–0.16, 3.65)	2.47 (0.91, 4.03)*	4.36 (1.66, 7.06)*	2.06 (–0.67, 4.78)	0.77 (–2.61, 4.14)	3.51 (–2.41, 9.43)	1.28 (–4.62, 7.19)
PNC_0.3__0–0__.35_	1.85 (0.84, 2.85)*	3.71 (1.96, 5.47)*	1.53 (–0.21, 3.26)	2.22 (0.77, 3.68)*	3.91 (1.28, 6.55)*	1.94 (–0.54, 4.41)	0.81 (–2.33, 3.96)	2.99 (–2.81, 8.79)	1.37 (–3.98, 6.72)
PNC_0.3__5–0__.40_	1.31 (0.52, 2.09)*	2.93 (1.39, 4.47)*	1.05 (–0.33, 2.44)	1.60 (0.47, 2.74)*	2.85 (0.54, 5.17)*	1.48 (–0.49, 3.45)	0.79 (–1.67, 3.26)	2.24 (–2.86, 7.33)	1.27 (–2.99, 5.53)
PNC_0.4__0–0__45_	0.69 (0.18, 1.21)*	2.29 (0.94, 3.64)*	0.55 (–0.41, 1.52)	0.92 (0.17, 1.66)*	2.01 (–0.03, 4.05)	0.87 (–0.51, 2.24)	0.72 (–0.88, 2.32)	2.15 (–2.31, 6.62)	1.00 (–1.96, 3.96)
PNC_0.4__5–0__.50_	0.45 (0.04, 0.87)*	2.11 (0.72, 3.49)*	0.37 (–0.45, 1.19)	0.64 (0.05, 1.23)*	1.67 (–0.43, 3.76)	0.63 (–0.54, 1.19)	0.66 (–0.60, 1.92)	2.53 (–2.03, 7.09)	0.85 (–1.65, 3.36)
PNC_0.5__0–0__.65_	0.36 (–0.04, 0.76)	2.33 (0.76, 3.91)*	0.23 (–0.49, 0.94)	0.59 (0.02, 1.17)*	1.83 (–0.54, 4.21)	0.49 (–0.53, 1.51)	0.66 (–0.57, 1.90)	3.37 (–1.79, 8.52)	0.68 (–1.51, 2.86)
PNC_0.65–1.0_	0.12 (–0.22, 0.45)	2.77 (0.85, 4.68)*	–0.02 (–0.76, 0.71)	0.37 (–0.10, 0.84)	2.23 (–0.66, 5.11)	0.34 (–0.70, 1.38)	0.42 (–0.59, 1.43)	4.85 (–1.25, 10.94)	0.40 (–1.83, 2.62)
PNC_1.__0–2__.5_	–0.12 (–0.43, 0.18)	3.68 (1.12, 6.23)*	–0.29 (–0.90, 0.32)	0.08 (–0.34, 0.51)	2.84 (–1.00, 6.68)	0.07 (–0.77, 0.92)	0.01 (–0.91, 0.90)	6.25 (–1.82, 14.32)	–0.19 (–1.99, 1.61)
PNC_2.5–10_	–0.03 (–0.08, 0.02)	2.45 (0.35, 4.55)*	–0.06 (–0.15, 0.04)	0.00 (–0.07, 0.06)	2.59 (–0.50, 5.68)	0.00 (–0.13, 0.13)	–0.03 (–0.17, 0.11)	4.46 (–2.23, 11.16)	–0.06 (–0.35, 0.22)
^***a***^Warm season, May–October. ^***b***^Cool season, November–April. *p < 0.05.									

Effect estimates for exposures during the warm season were approximately two times higher than corresponding estimates for the entire study period (Table 3). We did not observe statistically significant associations of PNCs and mortality for any particle size fractions during the cool season. For example, an IQR increase in PNC_0.25–0.28_ during the warm season (2,000 particles/cm^3^) was associated with 4.21% higher mortality (95% CI: 2.43, 5.99%), whereas the corresponding estimate for an IQR increase during the cool season (3,600 particles/cm^3^) was only 1.92% (95% CI: –0.14, 3.97%).

Associations with IQR increases in PNCs for particles < 0.40 μm remained statistically significant after adjustment for mass concentrations of PM_10_, PM_2.5_, SO_2_, and NO_2_ in two-pollutant models (Table 4). Associations with PNCs for particles < 0.50 μm were stronger when adjusted for PM_2.5–10_. Controlling for moving average temperature over the previous three days (lag 1–3), instead of temperature on the current day, had little impact on estimated associations with PNCs.

**Table 4:** Percent change (95% CI) of daily all-natural-cause mortality associated with a 2-day moving average IQR incremental change in particle fractions in Shenyang, China, adjusted for SO_2_, NO_2_, PM_10_, PM_2.5_, PM_2.5–10_, and temperature.

Particle fraction	Adjusted for					
	SO_2_	NO_2_	PM_10_	PM_2.5_	PM_2.5–10_	Temperature (lag 1–3)
PNC_0.2__5–0__.28_	2.04 (0.53, 3.54)*	1.66 (0.14, 3.17)*	1.75 (0.26, 3.24)*	2.18 (0.81, 3.55)*	2.52 (1.34, 3.71)*	2.05 (0.81, 3.29)*
PNC_0.2__8–0__.30_	1.79 (0.39, 3.18)*	1.42 (0.03, 2.82)*	1.57 (0.15, 2.98)*	1.86 (0.60, 3.13)*	2.20 (1.11, 3.29)*	1.78 (0.65, 2.91)*
PNC_0.3__0–0__.35_	1.56 (0.25, 2.87)*	1.20 (–0.11, 2.51)	1.38 (0.02, 2.74)*	1.57 (0.39, 2.75)*	1.94 (0.92, 2.96)*	1.56 (0.51, 2.61)*
PNC_0.3__5–0__.40_	1.05 (0.03, 2.06)*	0.72 (–0.30, 1.75)	0.89 (–0.16, 1.95)	0.97 (0.09, 1.86)*	1.36 (0.56, 2.17)*	1.09 (0.28, 1.90)*
PNC_0.4__0–0__.45_	0.49 (–0.17, 1.15)	0.26 (–0.40, 0.92)	0.39 (–0.29, 1.08)	0.42 (–0.14, 0.98)	0.73 (0.20, 1.26)*	0.57 (0.04, 1.09)*
PNC_0.4__5–0__.50_	0.27 (–0.24, 0.78)	0.09 (–0.43, 0.61)	0.21 (–0.33, 0.76)	0.22 (–0.22, 0.66)	0.47 (0.06, 0.89)*	0.36 (–0.06, 0.78)
PNC_0.5__0–0__.65_	0.16 (–0.34, 0.66)	–0.01 (–0.51, 0.50)	0.11 (–0.42, 0.65)	0.13 (–0.30, 0.55)	0.37 (–0.04, 0.79)	0.27 (–0.13, 0.68)
PNC_0.65–1.0_	–0.07 (–0.47, 0.33)	0.15 (–0.54, 0.25)	–0.12 (–0.56, 0.32)	–0.06 (–0.40, 0.29)	0.10 (–0.24, 0.44)	0.05 (–0.28, 0.39)
PNC_1.__0–2__.5_	–0.19 (–0.52, 0.14)	–0.18 (–0.50, 0.14)	–0.19 (–0.52, 0.14)	–0.16 (–0.47, 0.15)	–0.15 (–0.46, 0.17)	–0.15 (–0.46, 0.16)
PNC_2.5–10_	–0.04 (–0.08, 0.01)	–0.03 (–0.08, 0.02)	–0.03 (–0.08, 0.02)	–0.03 (–0.08, 0.02)	–0.03 (–0.08, 0.02)	–0.03 (–0.08, 0.01)
*p < 0.05.						

**Figure 1:**
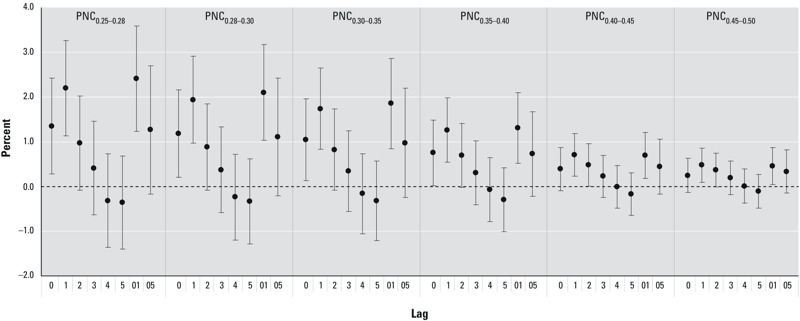
Percent increase (mean and 95% CI) of daily mortality associated with an IQR increase in PNCs of particles < 0.50 μm in diameter using different lag structures in Shenyang, China. Lags 0–5 represent single-day average exposures for the same day (lag 0) up to the fifth previous day (lag 5). Lags 01 and 05 represent 24-hour moving averages for the same day and previous day (lag 01) and the 6-day moving average for the same day through the previous 5 days (lag 05).

We observed similar trends for PNCs of 0.25–0.50 μm particles when different lag structures were modeled (Figure 1), with the highest effect estimates for single day lags on lag day 1, and no evidence of associations by lag day 5. Changing the df per year of calendar time from 7 df to 4–10 df did not substantially affect the association of PNCs with daily mortality (data not shown). Associations with broader categories of particle sizes (PNC_0.25–0.3_, PNC_0.3–0.5_, and PNC_0.5–1.0_) were consistent with findings for the smaller particle size groups used in the main analysis (see Supplemental Material, Table S3).

## Discussion

In this time-series study, we found significant positive associations between PNCs for particles from 0.25 to 0.5 μm in diameter and daily mortality that seemed to increase in magnitude as particle size decreased. Our estimates were relatively robust to adjustment for mass concentrations of PM_10_, PM_2.5_, PM_2.5–10_, SO_2_, and NO_2_, suggesting that PNCs may have independent effects on mortality. Associations between PNCs and daily mortality were much stronger in the warm season than in the cool season.

To our knowledge, ours is one of only a few studies to examine the health effects of PNCs in a developing country. Our results were consistent with previous studies conducted in Erfurt, Germany (Stolzel et al. 2007; Wichmann et al. 2000) and in Beijing, China (Breitner et al. 2011; Leitte et al. 2012). Wichmann et al. (2000) reported positive associations between daily mortality and PNCs for particles between 0.01 and 2.5 μm, whereas we observed significant associations only for particle fractions between 0.25 μm and 0.5 μm. Previous estimates of associations between mortality and lagged PNCs have been inconsistent. In our analysis, associations with single-day lags were strongest for lag day 1, and associations were significant for the first 3 lag days only. In contrast, other studies have reported evidence of more delayed effects for smaller particles (Ibald-Mulli et al. 2002; Leitte et al. 2012; Stolzel et al. 2007). For example, Leitte et al (2012) reported the strongest association between PNC_0.3–1.0_ and daily mortality using a lag of 4 days. More studies are needed to clarify the lag structure for PNCs of various sizes.

Although previous epidemiologic studies have reported short-term associations of PNCs with hospital admissions for respiratory disease in adults ≥ 65 years of age (Andersen et al. 2008) and pulmonary function in nonsmoking asthmatics (Peters et al. 1997), we did not observe significant positive associations between PNCs and respiratory mortality in our study population. The nonsignificant associations we observed for respiratory mortality, in contrast with significant positive associations with cardiovascular mortality, were consistent with findings reported in the study from Erfurt, Germany, where a sensitivity analysis suggested that associations between ultrafine particle PNCs and cardiorespiratory mortality were probably due primarily to associations with cardiovascular mortality (Stolzel et al. 2007). Significant associations between PNCs and respiratory outcomes reported by previous studies were for ultrafine particles (< 0.1 μm in diameter), whereas the smallest particles measured in the present study were 0.25 μm in diameter; therefore, it may be that ultrafine particles, but not larger particles, are responsible for respiratory effects. In addition, previous study populations included patients with asthma or chronic obstructive pulmonary disease who are assumed to be especially susceptible to air pollution (Zanobetti et al. 2000). Our study population, on the other hand, consisted of the general population in one city. Also, cardiovascular deaths due to short-term exposure are more likely than respiratory deaths (Basso et al. 1999), especially for those deaths related to air pollution (Dockery 2001). The daily number of deaths due to respiratory diseases is smaller than the number of deaths due to cardiovascular diseases, which limited our ability to estimate associations with respiratory mortality. Furthermore, it may be more informative to study associations of PNCs with respiratory morbidity and subclinical indicators of disease, instead of respiratory mortality.

The null association between PNCs of coarse particles (2.5–10 μm in diameter) and daily mortality in our analysis was consistent with results of studies focusing on mass concentration of coarse particles (Chen et al. 2011; Peng et al. 2008). However, because exposures to coarse particles are more variable than exposures to smaller particles within cities (Wilson and Suh 1997), our results for PNC_2.5–10_, which were based on data from a single monitoring site, should be interpreted with caution. In addition, because the exposure contrast for coarse particles (IQR of 4 particles/cm^3^) was relatively small (e.g., compared with an IQR of 2,600/cm^3^ for PNC_0.25–0.28_), associations between an IQR increase in coarse particles and mortality may have been more difficult to detect.

Associations between PNCs and daily mortality were stronger during the warm season than in the cool season. The pattern of exposure to ambient particles in populations may change from season to season. Because of the low temperature and the widespread use of central heating system in winter, residents of Shenyang generally stay home and close their windows as much as possible, thus reducing likelihood of exposure to outdoor air pollution. In contrast, the climate is more pleasant in the warm season; therefore, exposure to air pollution would likely be higher due to the increase in residents’ outdoor activities and greater penetration of outdoor particles into indoor environments from increased natural ventilation. Also, high temperatures in the summer might enhance adverse health effects of airborne particles (Meng et al. 2012). Alternatively, stronger associations observed in the warm season might relate to lower background mortality in summer, resulting in a larger pool of susceptible people (Nawrot et al. 2007).

Our study has limitations. We estimated associations for PNCs of particles in multiple size classes and lag times with three different mortality outcomes; some significant associations, therefore, may have occurred by chance. Due to limitations of the measuring equipment, we were unable to estimate exposures to UFPs. In addition, collinearity between PNCs and copollutants limited our ability to separate the independent effect for individual pollutants.

Exposure misclassification is a well-recognized limitation of time-series studies. We used the results from one fixed-site monitoring station as a proxy for population exposures to PNCs, and we were unable to assess spatial variation. However, high daily temporal correlations of PNCs among monitoring sites have been reported for several European cities (Buzorius et al. 1999; Cyrys et al. 2008; Puustinen et al. 2007), which suggests that using one carefully chosen monitoring site is a reasonable approach to characterize particle number concentrations for epidemiologic time-series studies. Our approach was consistent with previous studies of PNCs and mortality in Erfurt, Germany (Wichmann et al. 2000) and in Beijing, China (Leitte et al. 2012), which also relied on monitoring data from one carefully chosen site.

## Conclusions

Our analyses suggest that particle fractions measuring < 0.5 μm in diameter may be most responsible for adverse health effects linked with particulate air pollution. Associations between PNCs and mortality appeared to be independent of particle mass concentrations and exposures to other gas-pollutants, and they were much stronger during the warm season than the cool season. These findings support further exploration of the PNC-related health hazards, and may help inform the development of environmental policies to protect public health in China.

## Supplemental Material

(233 KB) PDF

## References

[r1] Andersen ZJ, Wahlin P, Raaschou-Nielsen O, Ketzel M, Scheike T, Loft S (2008). Size distribution and total number concentration of ultrafine and accumulation mode particles and hospital admissions in children and the elderly in Copenhagen, Denmark.. Occup Environ Med.

[r2] Basso C, Corrado D, Thiene G (1999). Cardiovascular causes of sudden death in young individuals including athletes.. Cardiol Rev.

[r3] Bell ML, Samet JM, Dominici F (2004). Time-series studies of particulate matter.. Annu Rev Public Health.

[r4] Breitner S, Liu L, Cyrys J, Bruske I, Franck U, Schlink U (2011). Sub-micrometer particulate air pollution and cardiovascular mortality in Beijing, China.. Sci Total Environ.

[r5] Brunekreef B, Forsberg B (2005). Epidemiological evidence of effects of coarse airborne particles on health.. Eur Respir J.

[r6] Buzorius G, Hämeri K, Pekkanen J, Kulmala M (1999). Spatial variation of aerosol number concentration in Helsinki City.. Atmos Environ.

[r7] Chen R, Kan H, Chen B, Huang W, Bai Z, Song G (2012). Association of particulate air pollution with daily mortality: the China Air Pollution and Health Effects Study.. Am J Epidemiol.

[r8] Chen R, Li Y, Ma Y, Pan G, Zeng G, Xu X (2011). Coarse particles and mortality in three Chinese cities: the China Air Pollution and Health Effects Study (CAPES).. Sci Total Environ.

[r9] Cyrys J, Pitz M, Heinrich J, Wichmann HE, Peters A (2008). Spatial and temporal variation of particle number concentration in Augsburg, Germany.. Sci Total Environ.

[r10] Delfino RJ Sioutas C Malik S 2005 Potential role of ultrafine particles in associations between airborne particle mass and cardiovascular health. Environ Health Perspect 113 934 946; 10.1289/ehp.7938 16079061 PMC1280331

[r11] Dockery DW (2001). Epidemiologic evidence of cardiovascular effects of particulate air pollution.. Environ Health Perspect.

[r12] Dominici F, Peng RD, Bell ML, Pham L, McDermott A, Zeger SL (2006). Fine particulate air pollution and hospital admission for cardiovascular and respiratory diseases.. JAMA.

[r13] Ibald-Mulli A, Wichmann HE, Kreyling W, Peters A (2002). Epidemiological evidence on health effects of ultrafine particles.. J Aerosol Med.

[r14] Kan H Chen B Hong C 2009 Health impact of outdoor air pollution in China: current knowledge and future research needs [Editorial] Environ Health Perspect 117 A187; 10.1289/ehp.12737 19478975 PMC2685855

[r15] Leitte AM, Schlink U, Herbarth O, Wiedensohler A, Pan XC, Hu M (2012). Associations between size-segregated particle number concentrations and respiratory mortality in Beijing, China.. Int J Environ Health Res.

[r16] Meng X, Zhang Y, Zhao Z, Duan X, Xu X, Kan H (2012). Temperature modifies the acute effect of particulate air pollution on mortality in eight Chinese cities.. Sci Total Environ.

[r17] Nawrot TS, Torfs R, Fierens F, De Henauw S, Hoet PH, Van Kersschaever G (2007). Stronger associations between daily mortality and fine particulate air pollution in summer than in winter: evidence from a heavily polluted region in western Europe.. J Epidemiol Community Health.

[r18] Pekkanen J, Peters A, Hoek G, Tiittanen P, Brunekreef B, de Hartog J (2002). Particulate air pollution and risk of ST-segment depression during repeated submaximal exercise tests among subjects with coronary heart disease: the Exposure and Risk Assessment for Fine and Ultrafine Particles in Ambient Air (ULTRA) study.. Circulation.

[r19] Peng RD, Chang HH, Bell ML, McDermott A, Zeger SL, Samet JM (2008). Coarse particulate matter air pollution and hospital admissions for cardiovascular and respiratory diseases among medicare patients.. JAMA.

[r20] Peters A, Wichmann HE, Tuch T, Heinrich J, Heyder J (1997). Respiratory effects are associated with the number of ultrafine particles.. Am J Respir Crit Care Med.

[r21] Puustinen A, Hämeri K, Pekkanen J, Kulmala M, De Hartog J, Meliefste K (2007). Spatial variation of particle number and mass over four European cities.. Atmos Environ.

[r22] Stolzel M, Breitner S, Cyrys J, Pitz M, Wölke G, Kreyling W (2007). Daily mortality and particulate matter in different size classes in Erfurt, Germany.. J Expo Sci Environ Epidemiol.

[r23] Valavanidis A Fiotakis K Vlachogianni T 2008 Airborne particulate matter and human health: toxicological assessment and importance of size and composition of particles for oxidative damage and carcinogenic mechanisms. J Environ Sci Health, Part C 26 339 362 10.1080/10590500802494538 19034792

[r24] Wichmann HE, Spix C, Tuch T, Wölke G, Peters A, Heinrich J (2000). Daily mortality and fine and ultrafine particles in Erfurt, Germany. Part I: role of particle number and particle mass.. Res Rep Health Eff Inst.

[r25] Wilson WE, Suh HH (1997). Fine particles and coarse particles: concentration relationships relevant to epidemiologic studies.. J Air Waste Manage Assoc.

[r26] Wong CM Vichit-Vadakan N Kan H Qian Z 2008 Public Health and Air Pollution in Asia (PAPA): a multicity study of short-term effects of air pollution on mortality. Environ Health Perspect 116 1195 1202; 10.1289/ehp.11257 18795163 PMC2535622

[r27] World Health Organization. (1992). International Statistical Classification of Diseases and Related Health Problems, Tenth Revision.

[r28] Zanobetti A, Schwartz J, Gold D (2000). Are there sensitive subgroups for the effects of airborne particles?. Environ Health Perspect.

[r29] Zeger SL, Irizarry R, Peng RD (2006). On time series analysis of public health and biomedical data.. Annu Rev Public Health.

