# Supplementary material for: Size-Fractionated Particle Number Concentrations and Daily Mortality in a Chinese City
Source: Environ Health Perspect. 2013 Aug 13;121(10):1174–8. doi: 10.1289/ehp.1206398 (PMC3801202; doi:10.1289/ehp.1206398)
Supplement: (233 KB) PDF [file ehp.1206398.s001.pdf]

Environmental Health Perspectives doi:10.1289/ehp.1206398

Supplemental Material

Size-Fractionated Particle Number Concentrations and Daily Mortality in a Chinese City

Xia Meng, Yanjun Ma, Renjie Chen, Zhijun Zhou, Bingheng Chen, and Haidong Kan

Table of contents

Supplemental Material, Table S1. Summary statistics of PNC in warm/cold seasons in Shenyang, China. -- 2

Supplemental Material, Table S2. Percent change (95%CI) in daily mortality associated with IQR and 10-ug/m^3 increases in 2-day moving average particle mass and gaseous pollutant concentrations in Shenyang, China. -- 3

Supplemental Material, Table S3. Percent change (95%CI) in daily mortality associated with an IQR increase in 2-day moving average particle fractions in Shenyang, China. ------------- 4

Supplemental Material, Table S1. Summary statistics of PNC in warm/cold seasons in Shenyang, China.[a]

Variables	Mean±SD	Minimum	Maximum	IQR
Warm Season				
$PNC_{0.25-0.28}$	2800±110	380	10000	2000
$PNC_{0.28-0.30}$	2100±86	260	8600	1600
$PNC_{0.30-0.35}$	1400±66	170	6800	1200
$PNC_{0.35-0.40}$	780±43	81	4700	680
$PNC_{0.40-0.45}$	350±22	28	2500	300
$PNC_{0.45-0.50}$	170±12	11	1400	160
$PNC_{0.50-0.65}$	150±9	9	1100	150
$PNC_{0.65-1.0}$	54±4	4	280	48
$PNC_{1.0-2.5}$	18±0.8	1.0	100	18
$PNC_{2.5-10}$	4±0.2	0.1	36	3
Cold Season				
$PNC_{0.25-0.28}$	4300±170	540	16000	3600
$PNC_{0.28-0.30}$	3300±150	380	15000	2800
$PNC_{0.30-0.35}$	2300±120	250	13000	2100
$PNC_{0.35-0.40}$	1300±88	140	9600	1200
$PNC_{0.40-0.45}$	670±59	42	6400	560
$PNC_{0.45-0.50}$	400±38	16	4300	320
$PNC_{0.50-0.65}$	390±39	12	4300	280
$PNC_{0.65-1.0}$	160±17	4	2000	120
$PNC_{1.0-2.5}$	46±7	1.0	1900	40
$PNC_{2.5-10}$	15±8	0.1	2600	8

[a] PNC are reported as particles / cm^3

Supplemental Material, Table S2. Percent change (95%CI) in daily mortality associated with IQR and 10-ug/m^3 increases in 2-day moving average particle mass and gaseous pollutant concentrations in Shenyang, China.

	For IQR incremental change			For 10 ug/m^3 incremental change		
	All natural causes	Cardiovascular	Respiratory	All natural causes	Cardiovascular	Respiratory
$PM_{2.5}$	2.75 (1.35, 4.15)*	2.65 (0.63, 4.68)*	4.33 (-0.05, 8.71)	0.43 (0.21, 0.65)*	0.42 (0.10, 0.73)*	0.68 (-0.01, 1.36)
PM_{10}	2.71 (1.37, 4.05)*	2.45 (0.50, 4.40)*	4.71 (0.46, 8.96)*	0.31 (0.16, 0.47)*	0.28 (0.06, 0.51)*	0.55 (0.05, 1.04)*
$PM_{10-2.5}$	1.04 (-0.30, 2.37)	0.92 (-1.01, 2.85)	2.05 (-2.27, 6.38)	0.28 (-0.08, 0.65)	0.25 (-0.27, 0.78)	0.56 (-0.62, 1.74)
SO_2	2.09 (0.33, 3.84)*	1.64 (-0.88, 4.17)	3.54 (-1.95, 9.03)	0.43 (0.07, 0.79)*	0.34 (-0.18, 0.85)	0.73 (-0.40, 1.85)
NO_2	3.49 (2.03, 4.95)*	3.26 (1.15, 5.38)*	6.75 (2.17, 11.32)*	2.09 (1.22, 2.97)*	1.96 (0.69, 3.23)*	4.05 (1.30, 6.80)*

* $p < 0.05$

Supplemental Material, Table S3. Percent change (95%CI) in daily mortality associated with an IQR increase in 2-day moving average particle fractions in Shenyang, China.

	All natural causes			Cardiovascular			Respiratory		
Particle fraction	All period	Warm	Cold	All period	Warm	Cold	All period	Warm	Cold
$PNC_{0.25\text{-}0.30}$	2.25 (1.13, 3.36)*	4.07 (2.32, 5.82)*	1.80 (-0.14, 3.75)	2.62 (1.00, 4.23)*	4.39 (1.77, 7.00)*	2.08 (-0.70, 4.87)	0.74 (-2.77, 4.24)	3.61 (-2.12, 9.35)	1.15 (-4.89, 7.19)
$PNC_{0.30\text{-}0.50}$	1.31 (0.52, 2.09)*	3.15 (1.55, 4.75)*	1.05 (-0.35, 2.44)	1.62 (0.49, 2.76)*	3.12 (0.72, 5.52)*	1.45 (-0.54, 3.44)	0.85 (-1.61, 3.31)	2.63 (-2.65, 7.91)	1.28 (-3.02, 5.58)
$PNC_{0.50\text{-}1.0}$	0.29 (-0.10, 0.68)	2.42 (0.80, 4.03)*	0.16 (-0.59, 0.91)	0.54 (-0.02, 1.10)	1.91 (-0.52, 4.34)	0.47 (-0.60, 1.54)	0.61 (-0.59, 1.80)	3.66 (-1.59, 8.91)	0.62 (-1.67, 2.91)

* P<0.05
